# Integrated Chemical Characterization, Network Pharmacology and Transcriptomics to Explore the Mechanism of Sesquiterpenoids Isolated from *Gynura divaricata* (L.) DC. against Chronic Myelogenous Leukemia

**DOI:** 10.3390/ph15111435

**Published:** 2022-11-19

**Authors:** Xinyuan Ye, Long Wang, Xin Yang, Jie Yang, Jie Zhou, Cai Lan, Fahsai Kantawong, Warunee Kumsaiyai, Jianming Wu, Jing Zeng

**Affiliations:** 1 School of Pharmacy, Southwest Medical University, Luzhou 646000, China; 2 School of Basic Medical Science, Southwest Medical University, Luzhou 646000, China; 3 Faculty Associated Medical Sciences, Department of Medical Technology, Chiang Mai University, Chiang Mai 50200, Thailand; 4 Education Ministry Key Laboratory of Medical Electrophysiology, Southwest Medical University, Luzhou 646000, China; 5 Key Medical Laboratory of New Drug Discovery and Druggability Evaluation, Southwest Medical University, Luzhou 646000, China; 6 Key Laboratory of Activity Screening and Druggability Evaluation for Chinese Materia Medica, Southwest Medical University, Luzhou 646000, China

**Keywords:** *Gynura divaricata* (L.) DC., chronic myelogenous leukemia, molecular networking, sesquiterpenoids, network pharmacology, RNA sequencing

## Abstract

Chronic myelogenous leukemia (CML) is a serious threat to human health, while drugs for CML are limited. Herbal medicines with structural diversity, low toxicity and low drug resistance are always the most important source for drug discoveries. *Gynura divaricata* (L.) DC. is a well-known herbal medicine whose non-alkaline ingredients (GD-NAIs) were isolated. The GD-NAIs demonstrated potential anti-CML activity in our preliminary screening tests. However, the chemical components and underlying mechanism are still unknown. In this study, GD-NAIs were tentatively characterized using UHPLC-HRMS combined with molecular networking, which were composed of 75 sesquiterpenoids. Then, the anti-CML activities of GD-NAIs were evaluated and demonstrated significant suppression of proliferation and promotion of apoptosis in K562 cells. Furthermore, the mechanism of GD-NAIs against CML were elucidated using network pharmacology combined with RNA sequencing. Four sesquiterpenoids would be the main active ingredients of GD-NAIs against CML, which could regulate PD-L1 expression and the PD-1 checkpoint pathway in cancer, PI3K/AKT, JAK/STAT, TGF-β, estrogen, Notch and Wnt signaling pathways. In conclusion, our study reveals the composition of GD-NAIs, confirms its anti-CML activity and elucidates their underlying mechanism, which is a potential countermeasure for the treatment of CML.

## 1. Introduction

Chronic myelogenous leukemia (CML) is a rare malignant proliferative hematopoietic disease [1]. The molecular hallmark of CML is the Philadelphia chromosome, which involves the fusion of the v-abl Abelson murine leukemia viral oncogene homologue 1 (ABL1) gene on chromosome 9 with the breakpoint cluster region (*BCR*) gene on chromosome 22 [1]. The *BCR-ABL1* oncoprotein induces the activation of tyrosine kinase (TK), stimulates multiple signaling pathways and alters the expression of genes/molecules, which ultimately induce CML [2,3]. These pathways include Janus kinase/signal transducer and activator of transcription (JAK/STAT), Hedgehog (Hh), transforming growth factor-β (TGF-β), mammalian forkhead transcription factors of the class O (FoxO), PD-L1 expression and PD-1 checkpoint pathway in cancer, peroxisome proliferator-activated receptor (PPAR), wingless-type MMTV integration site family (Wnt), mitogen-activated protein kinase (MAPK), phosphoinositide 3-kinases/protein kinase B (PI3K/AKT) and Notch signaling pathways [3,4,5,6,7]. Tyrosine kinase inhibitors (TKIs), a class of commonly used targeted drugs, are recognized as first-line treatment for CML. However, their side effects are obvious, such as the inability to completely eliminate leukemia stem cells, a high recurrence rate after drug withdrawal, drug resistance and others. Therefore, it is urgent to search for alternative medicines with lower toxicity and less drug resistance for the treatment of CML.

Phytomedicines have been widely explored for the treatment of cancers and have less toxicity and higher efficacy, which shown promising therapeutic potential in cancers by regulating multiple pathways [8]. More than half of approved anticancer agents are either natural compounds or derivatives derived from herbal medicine [9]. Sesquiterpenoids with the character of a C-15-hydrocarbon skeleton are derived from farnesyl pyrophosphate (FPP) [10], which play an important role in the field of natural medicinal chemistry. Its anti-leukemia activities have been studied, such as curcumol, pseudolaric acid B, dihydroartemisinin and eglerisine [11,12,13,14].

*Gynura divaricata* (L.) DC. (GD), known as Bai Bei San Qi in China and Daun Ngokilo in Thailand [15], is a widely used herbal medicine [16,17]. GD was approved as a new source of food by the Ministry of Health of the People’s Republic of China in 2010 [18]. GD is not only a popular vegetable but is also a common tea consumed for the prevention and treatment of hyperglycemia [19,20,21,22]. According to preliminary studies, GD has a variety of pharmacological activities, such as hypoglycemic effects [20,21], improvement of insulin resistance [19,22] and antihypertensive effects [23].

In our previous study, a strong cation exchange-based solid-phase extraction (SPE) method was developed for the separation of GD extracts [24]. The alkaline ingredients (tripeptide analogs) were retained on the SPE column due to ion interactions, and the non-alkaline ingredients (GD-NAIs) were eluted from the column immediately. The non-alkaline ingredients were collected, and their bioactivity was screened, which demonstrated potential anti-CML activity in preliminary experiments. However, the chemical components of the GD-NAIs and the underlying mechanism of GD-NAIs against CML are largely unknown.

In this study, GD-NAIs were tentatively characterized by ultrahigh-performance liquid chromatography–tandem high-resolution mass spectrometry (UHPLC-HRMS) combined with molecular networking (MN). The anti-CML activity of GD-NAIs was confirmed in K562 cells, and the underlying mechanism of GD-NAIs against CML was further elucidated by a combination of network pharmacology and RNA-sequencing (RNA-seq). Our study demonstrates that GD-NAIs may be an alternative agent for treating CML.

## 2. Results

### 2.1. Characterization of GD-NAIs by UHPLC-HRMS-MN

GNPS, a web-based mass spectrometry system, is a useful platform for characterizing natural products [25]. Compounds with similar structures were clustered in an MN provided by GNPS. Among the MN, one node represents one compound, the greater cosine value (0–1) between two adjacent nodes indicates that the structural similarity is higher, and the color of nodes indicates different properties. Compounds can be easily deduced by analyzing the fragmentation patterns of adjacent nodes, which facilitates the simultaneous characterization of abundant and trace analogs [26].

**Figure 1:**
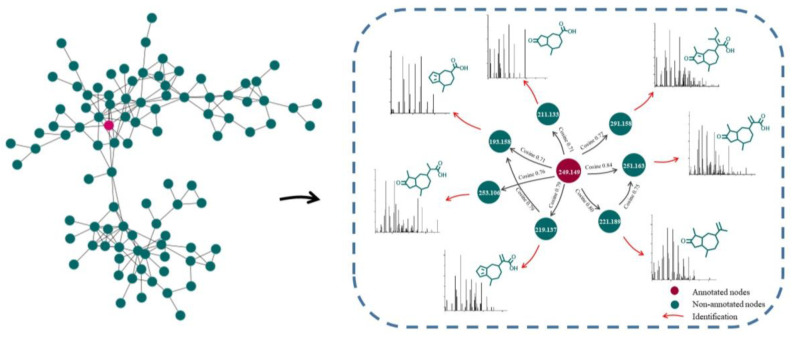
Molecular networking of non-alkaline ingredients isolated from *Gynura divaricata* (L.) DC. (GD-NAIs). The red node was identified as rupestonic acid (compound **1**) by GNPS library search, and the green nodes remain unknown.

**Figure 2:**
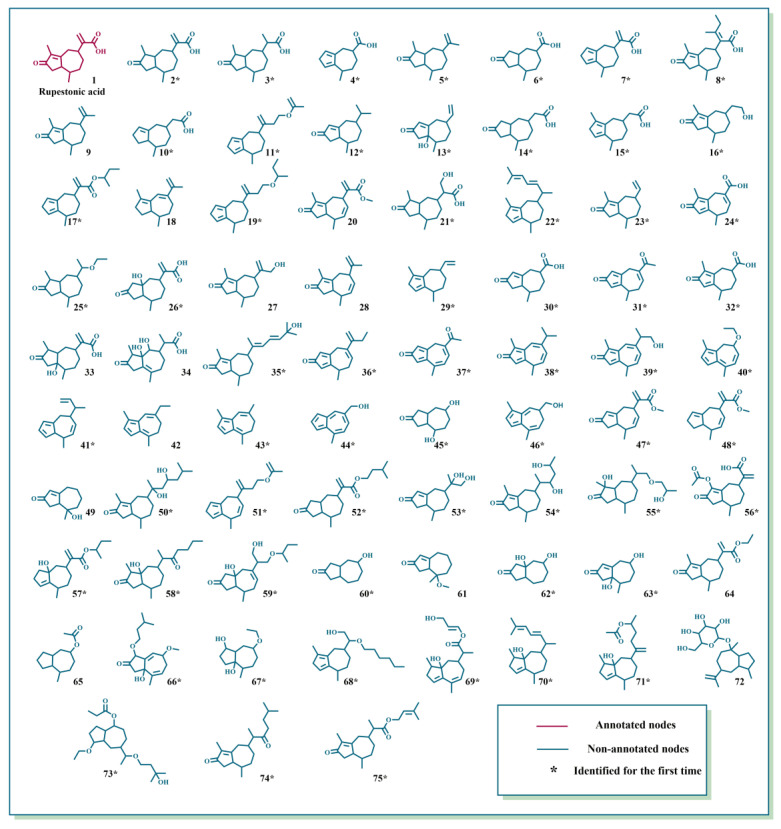
Structures of tentatively characterized sesquiterpenoids within *Gynura divaricata* (L.) DC.

Driven by advances in MN, UHPLC-HRMS combined with MN was used to tentatively characterize GD-NAIs. First, the MN of GD-NAIs was established (Figure 1). Red nodes represent compounds that were identified by GNPS library search, while green nodes represent unknown compounds. The results showed that the only red node was identified as a sesquiterpenoid rupestonic acid (compound **1**), yet others remain unknown. The clustered network indicated that these nodes (compounds) may be analogs of rupestonic acid that share similar fragmentation patterns. The fragmentation pattern of rupestonic acid was carefully studied and summarized. Thereafter, the connected compounds were tentatively characterized as a cluster of sesquiterpenoids, and a total of 75 compounds were tentatively characterized, of which 61 sesquiterpenoids were reported for the first time (Table 1 and Figure 2).

### 2.2. The Effect of GD-NAIs on Suppressing CML Cell Proliferation

**Figure 3:**
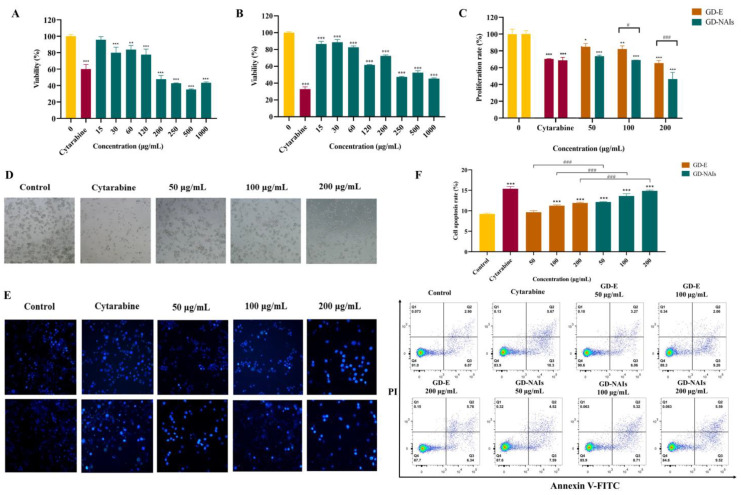
GD-NAIs suppress proliferation and induce apoptosis of K562 cells. (**A**) The viability of K562 cells was tested using a CCK-8 assay after treatment with cytarabine (20 nM) or GD-NAIs (15, 30, 60, 120, 250, 500 and 1000 μg/mL) for 48 h. (**B**) The viability of HEL cells was tested by CCK-8 assay after treatment with cytarabine (20 nM) or GD-NAIs (15, 30, 60, 120, 250, 500 and 1000 μg/mL) for 48 h. (**C**) The proliferation rate of K562 cells after treatment with cytarabine (20 nM) or GD-NAIs (50, 100 and 200 μg/mL) or GD-E (50, 100 and 200 μg/mL) for 48 h. (**D**) The cell morphology of K562 cells after treatment with cytarabine (20 nM) or GD-NAIs (50, 100 and 200 μg/mL) for 24 h. (**E**) Cell apoptosis was determined by Hoechst 33258 assay after treatment with cytarabine (20 nM) or GD-NAIs (50, 100 and 200 μg/mL) for 24 and 48 h. (**F**) Apoptosis was detected by flow cytometry after treatment with GD-NAIs (50, 100 and 200 μg/mL) or GD-E (50, 100 and 200 μg/mL) for 48 h, *n* = 3. * *p* < 0.05, ** *p* < 0.01, *** *p* < 0.001 compared to the control group. ^#^ *p* < 0.05, ^###^ *p* < 0.001 compared between two groups.

First, the cytotoxicity of GD-NAIs on L02 normal liver cells was evaluated by MTT assay. The results showed that the IC_50_ value of GD-NAIs was more than 1 mg/mL. Then, the effect of GD-NAIs at safe concentrations on K562 cells was assessed. The results demonstrated that GD-NAIs (30, 60, 120, 250, 500 and 1000 μg/mL) significantly inhibited the viability of K562 cells (Figure 3A). The inhibitory effect of GD-NAIs was also assessed in the acute erythroid leukemia (AEL) cell line HEL. The results demonstrated that GD-NAIs (15, 30, 60, 120, 250, 500 and 1000 μg/mL) were able to inhibit the viability of HEL cells (Figure 3B). As a positive drug for CML, cytarabine (20 nM) showed an obvious inhibitory effect on the viability of K562 and HEL cells (Figure 3A,B). Furthermore, the effect of GD-NAIs and GD-E on the proliferation of K562 cells was measured by CCK-8 assay. The results indicated that GD-NAIs and GD-E (50, 100 and 200 μg/mL) conspicuously inhibited the proliferation of K562 cells in a concentration-dependent manner (Figure 3C). The inhibitory ability of GD-NAIs on the proliferation of K562 cells at a concentration of 200 μg/mL was much stronger than that of cytarabine (20 nM) (Figure 3C). Besides, the inhibitory ability of GD-NAIs (100 and 200 μg/mL) was stronger than that of GD-E (Figure 3C). These results indicated that GD-NAIs are able to suppress the proliferation of K562 cells in a concentration-dependent manner.

### 2.3. The Effect of GD-NAIs on Inducing Cell Apoptosis

One of the hallmarks of CML is the deregulation of apoptosis [39,40]. Therefore, the effect of GD-NAIs on the apoptosis of K562 cells was verified. The cell morphology was observed and showed that the number of K562 cells in the GD-NAIs (50, 100 and 200 μg/mL)-treated and cytarabine (20 nM)-treated groups was markedly less than that of the control group (Figure 3D). GD-NAIs (50, 100 and 200 μg/mL) and cytarabine (20 nM) caused bursts and the death of K562 cells (Figure 3D). The Hoechst 33258 assay further demonstrated that many bright blue cells appeared in the GD-NAIs (50, 100 and 200 μg/mL)-treated and cytarabine (20 nM)-treated groups but seldom in the control group (Figure 3E), indicating that GD-NAIs could induce apoptosis of K562 cells. In addition, Annexin V-FITC/PI staining showed that the percentage of apoptotic cells in the GD-NAIs (50, 100 and 200 μg/mL)-, GD-E (50, 100 and 200 μg/mL)-treated and cytarabine (20 nM)-treated groups was much higher than that in the control group (Figure 3F). Moreover, the proapoptotic ability of GD-NAIs was stronger than that of GD-E (Figure 3F). Taken together, these results indicated that GD-NAIs can significantly promote apoptosis of K562 cells.

### 2.4. Network Pharmacology Analysis of GD-NAIs against CML

#### 2.4.1. Target Prediction and Screening of GD-NAIs against CML

**Figure 4:**
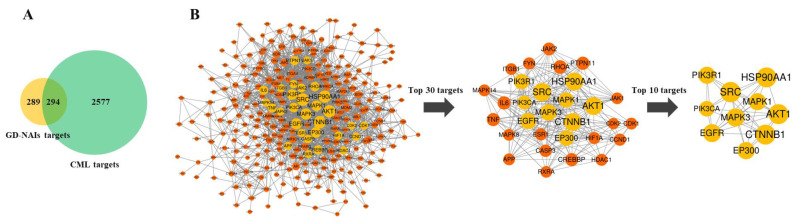
(**A**) The Venn diagram of the intersection of GD-NAIs targets and CML targets. (**B**) Protein–protein interaction (PPI) network of targets of GD-NAIs against CML.

To predict the mechanism of GD-NAIs against CML, network pharmacology was carried out. First, a total of 68 components of GD-NAIs were screened out using absorption and drug-likeness parameters (Table S1 (Supplementary Materials)). Then, 583 gene targets of GD-NAIs were obtained from the SwissTargetPrediction network database, and 2871 gene targets of CML were collected through OMIM, GeneCards, PharmGKB, Drugbank and Therapeutic Target Database (Figure 4A). A total of 294 overlapping genes were considered potential targets of GD-NAIs against CML (Figure 4A; Table S2).

#### 2.4.2. PPI Network of Targets of GD-NAIs against CML

To investigate the interactions between the targets of GD-NAIs against CML, 294 genes were imported into STRING to construct a PPI network. The results showed that the PPI network contained 277 nodes and 1823 edges with an average degree of 12.4 (Figure 4B). Among the PPI network, each node represented a gene target; the larger the node size was, the greater the degree value. The interaction strength was expressed by the number of node connections. The topological analysis of the PPI network based on the network parameter of degree was applied to screen out the key targets. The 57 targets’ values were higher than the two-fold median value (18). The top 30 targets based on the three-fold median value were selected (Figure 4B), which were tentatively regarded as the key targets of GD-NAIs against CML. A further screening parameter was set as the five-fold median value; thus, the top 10 targets including SRC, HSP90AA1, CTNNB1, AKT1, MAPK3, EGFR, EP300, PIK3R1, MAPK1 and PIK3CA were screened out (Figure 4B). These targets might be the key targets of GD-NAIs against CML.

#### 2.4.3. KEGG Pathway Enrichment Analysis of Targets of GD-NAIs against CML

**Figure 5:**
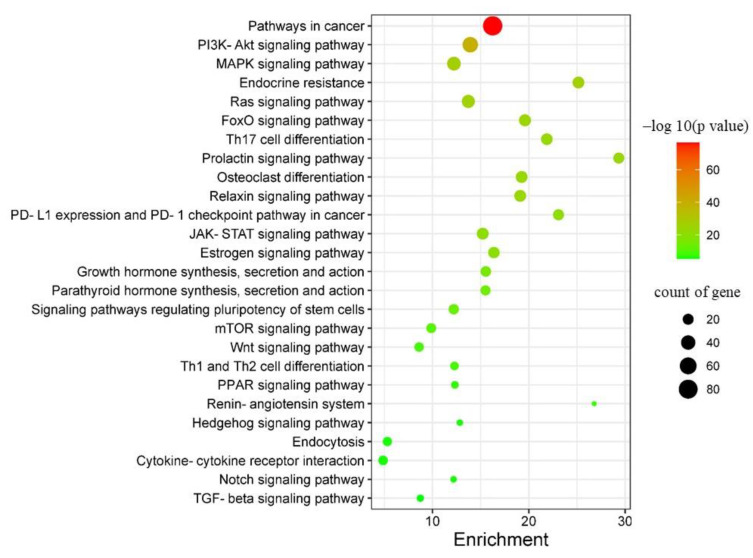
The KEGG pathways associated with GD-NAIs against CML.

KEGG pathway enrichment analysis of the 294 overlapping genes was applied to predict the signaling pathways regulated by GD-NAIs. The results revealed that the targets of GD-NAIs were mainly enriched in PI3K/AKT, MAPK, Endocrine resistance, Ras, FoxO, Th17 cell differentiation, JAK/STAT and TGF-β signaling pathways (Figure 5; Table S3), which are all potential therapeutic targets of CML.

#### 2.4.4. Compound-Target-Pathway Network and Analysis

**Figure 6:**
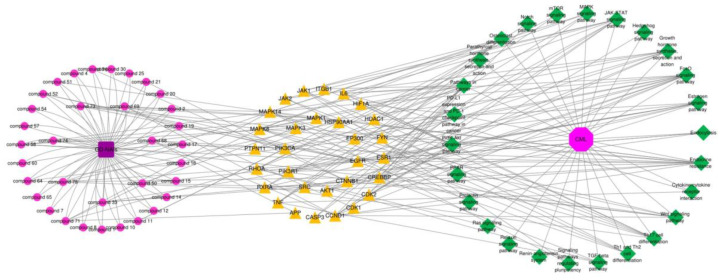
The compound–target–pathway network. Red circle nodes represent the components of GD-NAIs, yellow triangle nodes represent the targets, and green diamond modes represent the KEGG pathway.

Due to the great number of targets, the top 30 targets of GD-NAIs against CML were used to construct a compound–target–pathway network. The results showed that 92 nodes and 488 edges made up the network, which included 1 herbal medicine, 34 compounds, 30 targets, 26 pathways and 1 disease (Figure 6). The greater the degree value, the more nodes were connected to this node, which proved that the node contributed significantly to the network. Thirty-four components were related to the top 30 targets (Figure 6). Compounds **68**, **71**, **69** and **75** had large degree values (Figure 6), indicating that these compounds might be the potential active components of GD-NAIs in the treatment of CML.

### 2.5. Gene Expression Profile Regulated by GD-NAIs

**Figure 7:**
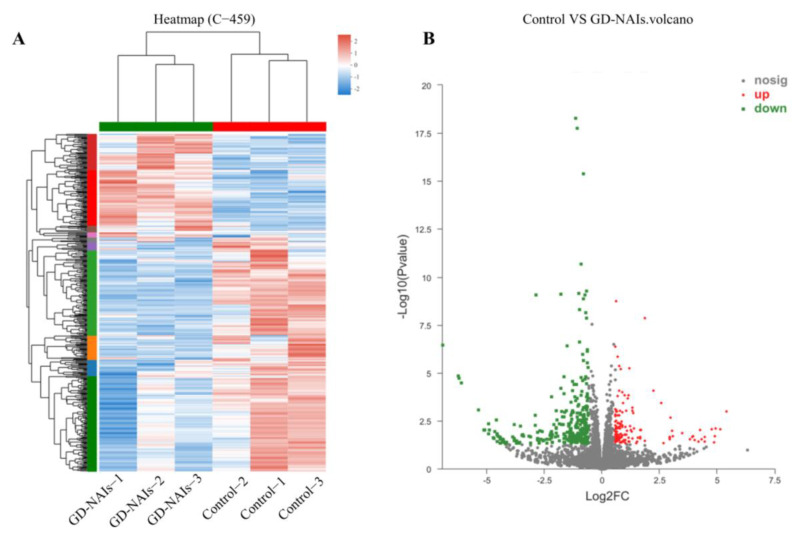
Gene expression profile induced by GD-NAIs. (**A**) Hierarchical clustering analysis of DEGs between the control and GD-NAIs-treated groups. (**B**) Volcano plot of DEGs between the control and GD-NAIs-treated groups.

**Figure 8:**
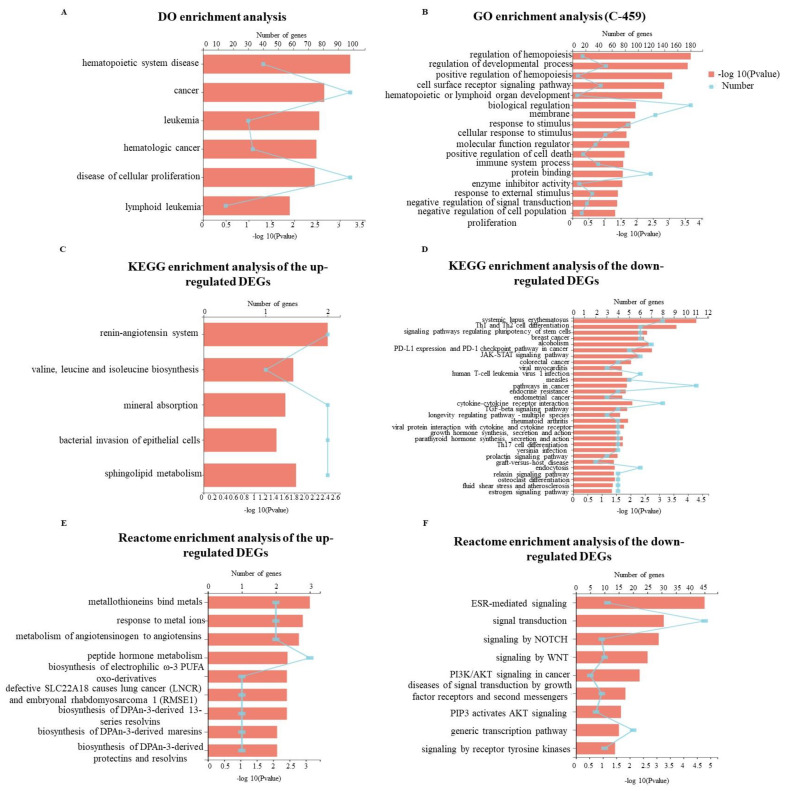
DO, GO, KEGG and Reactome enrichment analysis of DEGs induced by GD-NAIs. (**A**) DO enrichment analysis of DEGs. (**B**) GO enrichment analysis of DEGs. (**C**) KEGG enrichment analysis of the upregulated DEGs. (**D**) KEGG enrichment analysis of the downregulated DEGs. (**E**) Reactome enrichment analysis of the upregulated DEGs. (**F**) Reactome enrichment analysis of the downregulated DEGs.

To elucidate the underlying mechanism of GD-NAIs in inhibiting proliferation and inducing apoptosis of CML cells, RNA-seq was performed after K562 cells were treated with GD-NAIs (200 μg/mL) for 48 h. Hierarchical clustering analysis showed that there was a large gene expression difference between the control and GD-NAIs-treated groups (Figure 7A). Volcano plot statistics and the graph of DEGs revealed that there were 141 DEGs upregulated by GD-NAIs and 318 DEGs downregulated by GD-NAIs (Figure 7B), suggesting that GD-NAIs mainly played an inhibitory function on gene expression in K562 cells. DO enrichment analysis suggested that DEGs regulated by GD-NAIs were mainly involved in hematopoietic system disease, cancer, leukemia, hematologic cancer, cellular proliferation and lymphoid leukemia (Figure 8A), which was in line with the therapeutic action of GD-NAIs on CML. GO enrichment analysis showed that DEGs were markedly enriched in positive regulation of hemopoiesis, hematopoietic or lymphoid organ development, negative regulation of signal transduction, and negative regulation of cell population proliferation (Figure 8B). KEGG pathway enrichment analysis demonstrated that the upregulated DEGs were significantly associated with the renin-angiotensin system, valine, leucine and isoleucine biosynthesis, sphingolipid metabolism and soon (Figure 8C). Conversely, the downregulated DEGs were prominently enriched in PD-L1 expression and the PD-1 checkpoint pathway in cancer, pathways in cancer, TGF-β, estrogen-signaling pathways and so on (Figure 8D), which were all targets against CML. Reactome enrichment analysis revealed that the upregulated DEGs were mainly involved in metallothioneins-binding metals, the response to metal ions, metabolism of angiotensinogen to angiotensins, the biosynthesis of DPAn-3-derived 13-series resolvins and so on (Figure 8E). However, the downregulated DEGs were mostly enriched in ESR-mediated signaling, signaling by Notch, signaling by Wnt, PI3K/AKT signaling in cancer, signaling by receptor tyrosine kinases and so on, which were all targets for therapeutic intervention (Figure 8F). Combining the network pharmacology analysis, GO, KEGG and Reactome enrichment analysis, PD-L1 expression and the PD-1 checkpoint pathway in cancer, JAK/STAT, TGF-β, estrogen, Notch and Wnt were the main signaling pathways regulated by GD-NAIs for the treatment of CML.

## 3. Discussion

**Figure 9:**
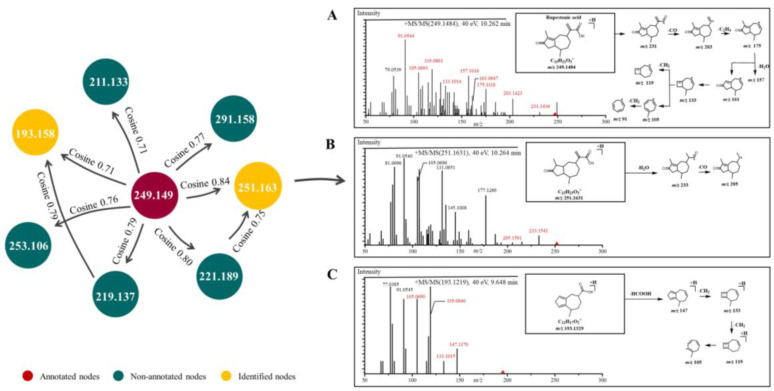
Mass spectrum and proposed fragmentation patterns of rupestonic acid (**A**), compound **2** (**B**) and compound **4** (**C**).

The development of CML is associated with multiple biological processes and signaling cascades [4]. Treatment of CML with herbal medicines has multitarget, multi-pathway and overall coordination characteristics. In the present study, we first tentatively characterized the chemical ingredients in GD-NAIs. The red node identified as rupestonic acid (compound **1**) via library search (Figure 9A) produced a precursor ion at *m*/*z* 249.1484 (−0.5 ppm, C_15_H_20_O_3_) and diagnostic ions at *m*/*z* 231.1436 ([M+H–H_2_O]^+^), 203.1423 ([M+H–H_2_O–CO_2_]^+^), 175.1118 ([M+H–H_2_O–CO_2_–C_2_H_4_]^+^), 161.0947 ([M+H–H_2_O–CO_2_–C_2_H_4_–CH_2_]^+^), 157.1016 ([M+H–H_2_O–CO_2_–C_2_H_4_–H_2_O]^+^), 133.1014 ([C_10_H_13_]^+^), 119.0861 ([C_9_H_11_]^+^), 105.0693 ([C_8_H_9_]^+^), 91.0544 ([C_7_H_7_]^+^), 81.0697 ([C_6_H_8_+H]^+^), 79.0539 ([C_6_H_6_+H]^+^), in which *m*/*z* 105.0693, 91.0544, 81.0697 and 79.0539 were specific ions of methyl-cycloheptane of rupestonic acid. Thereafter, a detailed analysis of the analogs was performed referring to the fragmentation rules of compound **1** as a seed node.

Compound **2** produced a precursor ion at *m*/*z* 251.1631 (−4.3 ppm, C_15_H_22_O_3_), which was 2 Da higher in mass than rupestonic acid (compound 1) and yielded the same product ions of methyl cycloheptane. In addition, the characteristic ions at *m*/*z* 233.1541 and 205.1591 corresponded to the successive loss of H_2_O and CO. Therefore, compound **2** was tentatively identified (Figure 9B). Compound **3** exhibited a precursor ion at *m*/*z* 253.1788 (−4.0, C_15_H_24_O_3_) and was then rapidly identified in a similar way (Figure S1). Compound **4** was tentatively characterized and exhibited a precursor ion at *m*/*z* 193.1219 (−2.1 ppm, C_12_H_16_O_2_) and specific ions at *m*/*z* 147.1170 ([M+H–HCOOH]^+^), 133.1015, 119.0840 and 105.0690 (Figure 9C). Similar MS/MS fragmentations were also observed in these four neighboring compounds (**5**, **6**, **7** and **8**) (Figures S2–S5). Other compounds were subsequently tentatively characterized based on the above-mentioned fragmentation patterns. Not surprisingly, these tentatively characterized compounds were all sesquiterpenoids.

Sesquiterpenoids are one of the most attractive components in natural product chemistry [9]. There are hundreds of natural sesquiterpenoids. Studies have confirmed that sesquiterpenoids have inhibitory effects on leukemia [41,42,43]. Several examples are given, including that curcumol isolated from *Curcuma longa* L. can induce the differentiation and apoptosis of CML cells by blocking the BCR/ABL-JAK2/STAT3 and PI3K/AKT-JNK signaling pathways and activating the BH3-only gene [11]. Pseudolaric acid B derived from *Pseudolarix kaempferi* was able to induce cell apoptosis in acute promyelocytic leukemia HL-60 cells by inhibiting tubulin polymerization, preventing cell division and activating caspase-3 [12]. Dihydroartemisinin significantly induced apoptosis and inhibited vascular endothelial growth factor (VEGF) expression in K562 cells [13]. We tentatively characterized 61 new sesquiterpenoids and 14 known sesquiterpenoids from GD-NAIs, and no pharmacological activity against CML of these ingredients has been reported, which needs to be further explored.

CML is characterized by the loss of proliferation control [6]. We thus monitored the effect of GD-NAIs on the proliferation of K562 cells and observed that GD-NAIs conspicuously suppressed the proliferation of K562 cells in a concentration-dependent manner. Another hallmark of CML is a defect in apoptosis [39,40]. We found that GD-NAIs could induce apoptosis of K562 cells. These results indicated that GD-NAIs were able to suppress the proliferation and promote apoptosis of CML cells, which is consistent with previous studies showing that sesquiterpenoids have an inhibitory effect on CML [11,13]. Furthermore, we have experimentally proved that GD-NAIs demonstrate stronger proliferation inhibition and proapoptotic ability on K562 cells than that of GD-E. Meanwhile, we demonstrated that GD-NAIs had low toxicity to normal liver cells, indicating that GD-NAIs have high potential for clinical application.

**Figure 10:**
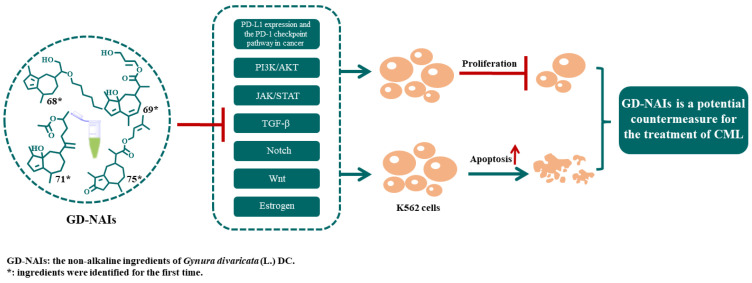
GD-NAIs exhibit anti-CML activity by targeting multiple pathways, including PD-L1 expression and the PD-1 checkpoint pathway in cancer, PI3K/AKT, JAK/STAT, TGF-β, estrogen, Notch and Wnt signaling pathways.

Subsequently, the pharmacological mechanism of GD-NAIs against CML was investigated through a combination of network pharmacology and RNA-seq. We proved that GD-NAIs can exert an inhibitory effect on CML by regulating multiple signaling pathways, including PD-L1 expression and the PD-1 checkpoint pathway in cancer, the PI3K/AKT, JAK/STAT, TGF-β, estrogen, Notch and Wnt signaling pathways. PD-L1 expression and the PD-1 checkpoint pathway in the cancer signaling pathway plays an important role in tumor immunity. PD-1 binds to its ligand PD-L1 and inhibits T-cell activity. Abnormally high PD-LI expression on tumor cells mediates tumor immune escape [44]. An efficient therapeutic strategy for CML is to block this signaling pathway [3]. The PI3K/AKT signaling pathway regulates a large number of cellular processes, such as the cell cycle, proliferation, differentiation and apoptosis [5,7,45], whose dysregulation in leukemia stem cells (LSCs) increases ROS production and promotes the survival of LSCs and their drug resistance [6]. Furthermore, dysregulation of the JAK/STAT signaling pathway causes proliferation and resistance to apoptosis, which is a hallmark of BCR-ABL-transformed CML cells [46]. It has been reported that LSCs have a self-renewal capacity to generate leukemia progenitor cells by activating the TGF-β, Notch and Wnt signaling pathways [6]. The TGF-β signaling pathway participates in a wide range of cellular processes, such as growth, proliferation and apoptosis [47]. It was efficient to inhibit this pathway to decrease the maintenance of LSCs and eliminate CML leukemia-initiating cells [48]. According to the literature, inhibiting the Notch signaling pathway has anti-CML activity [49]. Moreover, inhibiting the estrogen signaling pathway induces apoptosis in a variety of leukemia cells [50]. As predicted by network pharmacology, compounds **68**, **71**, **69**, **75**, etc., might be the main active components of GD-NAIs for the treatment of CML. Compound **68** might exert an inhibitory effect on CML through the regulation of the PI3K/AKT, JAK/STAT and Wnt signaling pathways. Compound **71** might inhibit the TGF-β and Wnt signaling pathways. Compound **69** could target the JAK/STAT and estrogen signaling pathways. Moreover, compound **75** inhibited the PI3K/AKT, TGF-β, Notch and Wnt signaling pathways. These studies and our findings suggest that GD-NAIs exhibit anti-CML activity by targeting multiple pathways, including PD-L1 expression and the PD-1 checkpoint pathway in cancer, PI3K/AKT, JAK/STAT, TGF-β, estrogen, Notch and Wnt signaling pathways (Figure 10).

## 4. Materials and Methods

### 4.1. Cell Culture

The cell lines (K562, HEL and L02) were purchased from American Type Culture Collection (Bethesda, MD, USA). The K562 and HEL cells were cultured in RPMI 1640 medium (Gibco, Thermo Fisher Scientific, Waltham, MA, USA) with 10% FBS (Gibco, Thermo Fisher Scientific, Waltham, MA, USA) and 100 U/mL penicillin-streptomycin (Beyotime, Shanghai, China). L02 cells were cultured in DMEM (Gibco, Thermo Fisher Scientific, Waltham, MA, USA). Cells were maintained at 37 °C with a 5% CO_2_ atmosphere.

### 4.2. Chemicals and Materials

GD collected from Luxian (105^◦^ 32′ 26.4′′ N; 28^◦^ 59′ 54.3′′ E) was authenticated by Professor Can Tang of the Department of Chinese Materia Medica, Southwest Medical University, China. The voucher specimen (SWMU-211205005) was deposited at the specimen repository of the Department of Traditional Chinese Medicine, Southwest Medical University. LC-MS grade acetonitrile and formic acid were purchased from Fisher Scientific (Waltham, MA, USA). Ethanol (Titan Scientific, Shanghai, China), methanol (Jinshan HuaShi, Chengdu, China), disodium hydrogen phosphate anhydrous (KeShi, Chengdu, China), and hydrochloric acid (XiLong Scientific, Chengdu, China) were used for isolation and enrichment of GD-NAIs. A Milli-Q water purification system (Billerica, MA, USA) was used to generate ultrapure water. Cytarabine was procured from Energy Chemical (Anqing, China).

### 4.3. Preparation of GD-NAIs

Samples were prepared according to a previously published method [24]. Whole fresh GD plants (1 kg) were prepared by the impregnation method with 20 L methanol–water (50:50, v v^−1^) with 0.1% hydrochloric acid for two weeks. After filtration, concentration and lyophilization, the extracts of GD (GD-E) were collected for further processing.

GD-E were prepared in ethanol–water (20:80, v v^−1^) containing 0.1% hydrochloric acid. A strong cation exchange silica-based solid-phase extraction (SPE) column (Acchrom, Beijing, China) was preconditioned with methanol and methanol–water (20:80, v v^−1^) with 250 mmol/L Na_2_HPO_4_. GD-E were loaded; thereafter, the column was eluted with 3 bead volumes of methanol–water (50:50, v v^−1^), and the GD-NAIs eluted immediately due to their nonionic properties. Finally, GD-NAIs were collected and lyophilized for further usage.

### 4.4. UHPLC-HRMS Method, Data Acquisition and Preprocessing

UHPLC-HRMS was used to characterize GD-NAIs. An UHPLC (Exion; Sciex) equipped with an Inertsil C_18_ column (100 × 2.1 mm, 3 μm) at 40 °C was employed. Formic acid (0.1%) in water (A) and acetonitrile (B) were used as eluents, and gradient elution was performed at a flow rate of 0.3 mL min^−1^. A gradient elution program was set as follows: 5% B at 0–2 min; 5–70% B at 2–18 min; 70–100% B at 18–20 min; and 100% B at 20–25 min. An X500R Q-TOF mass spectrometer (SCIEX, Framingham, MA, USA) equipped with an electrospray ionization (ESI) source was employed to analyze the eluate from UHPLC. The optimized MS and MS/MS parameters were set as follows: temperature: 500 °C; ion source gas 1 and 2:50 psi; curtain gas: 35 psi; CAD gas: 7 psi; collision energy: 40 ± 15 V; maximum candidate ions: 10; intensity threshold: 400 cps; full scan mass range: 100–1500 Da; and ion spray voltage: 5500 V.

Before uploading to the global natural products social (GNPS) molecular network web server (http://gnps.ucsd.edu (accessed on 1 January, 2021)), raw UHPLC-HRMS data files were prepared by ProteoWizard’s msConvert [51]. The precursor ion mass tolerance and fragment ion mass tolerance were set as ± 0.02 Da on the GNPS platform, and the other parameters were set as default values.

### 4.5. Cell Viability Assay

K562, HEL and L02 cells were cultured in 96-well plates at concentrations of 4 × 10^4^, 4 × 10^4^ and 2 × 10^4^ cells/mL (100 μL/well), respectively. Different concentrations of GD-NAIs (15, 30, 60, 120, 250, 500 and 1000 μg/mL) and cytarabine (20 nM) were added to each well. After 48 h of incubation at 37 °C, thiazolyl blue tetrazolium bromide (MTT) solution (Invitrogen, Thermo Fisher Scientific, Waltham, MA, USA) or Cell Counting Kit-8 (CCK-8) (GLPBIO, Montclair, CA, USA) was added to each well and incubated for 4 h at 37 °C. Absorbance was determined using a multi-well plate reader (PEIOU, Shanghai, China). The percentage of cell viability was calculated according to Equation (1):

(1) $$Viability\;\left(\%\right)=\frac{\mathrm{absorbance}\;\mathrm{of}\;\mathrm{sample}}{\mathrm{absorbance}\;\mathrm{of}\;\mathrm{blank}}\times 100$$

### 4.6. Cell Proliferation Assay

The proliferation of K562 cells was determined by MTT assay. Cells were treated with cytarabine (20 nM) or GD-NAIs (50, 100 and 200 μg/mL) or GD-E (50, 100 and 200 μg/mL) for 48 h. CCK-8 solution was added, and the absorbance was determined at 450 nm.

### 4.7. Cell Apoptosis Assay

BD Pharmingen™ FITC Annexin V Apoptosis Detection Kit I (BD Biosciences, San Jose, CA, USA) was used to detect apoptosis. Cells were treated with cytarabine (20 nM) or GD-NAIs (50, 100 and 200 μg/mL) or GD-E (50, 100 and 200 μg/mL), respectively. After 48 h, the cells were harvested and washed with cold phosphate-buffered saline (PBS). The samples were incubated with Annexin V-FITC and propidium iodide (PI) for 15 min at 37 °C in the dark. Cell apoptosis was measured using a BD FACSCanto II flow cytometer (BD Biosciences, San Jose, CA, USA)

In addition, the apoptosis of K562 cells was determined by Hoechst 33258 assay. After treatment with GD-NAIs (50, 100 and 200 μg/mL) for 24 and 48 h, the cells were harvested and washed with cold PBS. The supernatants were discarded, and the cells were resuspended in cell stain buffer. Thereafter, 5 μL of Hoechst 33258 stain (Invitrogen, Thermo Fisher Scientific, Waltham, MA, USA) was added to the cells and incubated for 15 min at 37 °C. Cryogenic centrifugation was performed to remove the staining solution. Eventually, the samples were photographed on a fluorescence microscope (Olympus, Tokyo, Japan) at a magnification of ×200. In addition, the cells were photographed using light microscopy (Nikon, Tokyo, Japan).

### 4.8. Network Pharmacology Analysis

#### 4.8.1. Prediction of Targets of GD-NAIs

Based on the tentatively characterized components in Section 4.4, the SwissADME network database (http://www.swissadme.ch/ (accessed on 15 January 2022)) [52] was employed to screen active components based on absorption and drug-likeness parameters. With the help of the SwissTargetPrediction network database (http://www.swisstargetprediction.ch/ (accessed on 15 January 2022)) [53], component-related gene targets were retrieved and duplicate genes were removed. The screening condition of genes was probability >0.1.

#### 4.8.2. Collection of Predicted Targets for CML

CML-related gene targets were prepared using key words such as “chronic myelogenous leukemia” and “Chronic myeloid leukemia” from OMIM (https://omim.org/ (accessed on 8 May, 2022)) [54], GeneCards (https://www.genecards.org/ (accessed on 8 May 2022)) [55], PharmGKB (https://www.pharmgkb.org/ (accessed on 8 May 2022)) [56], Drugbank (https://go.drugbank.com/ (accessed on 8 May 2022)) [57] and Therapeutic Target Database (http://db.idrblab.net/ttd/ (accessed on 8 May 2022)) [58]. Non-human genes were eliminated.

#### 4.8.3. Construction of Protein–Protein Interaction (PPI) Network

The gene targets in Section 4.8.1 and Section 4.8.2 were imported into Venny (http://www.liuxiaoyuyuan.cn/ (accessed on 8 May 2022)) [59]. The overlapping genes were regarded as targets of GD-NAIs against CML. STRING (https://cn.string-db.org/ (accessed on 8 May 2022)) [60] was used to construct the PPI network of targets of GD-NAIs against CML. The organism was set as “Homo sapiens”, the minimum required interaction score was set as 0.700, and disconnected nodes in the network were hidden. Cytoscape 3.7.2 was used for topological analysis of nodes to select the key targets in the PPI network. The threshold value of the first screening was set as the two-fold median value and the further screening parameter was set as the five-fold median value.

#### 4.8.4. Kyoto Encyclopedia of Genes and Genomes (KEGG) Pathway Enrichment Analysis

The Metascape database (https://metascape.org/gp/index.html#/main/step1 (accessed on 9 May 2022)) [61] was used for KEGG pathway enrichment analysis. A total of 294 overlapping genes of GD-NAIs and CML were uploaded to Metascape. Parameters were default values. The KEGG results were visualized using Cytoscape 3.7.2.

#### 4.8.5. Construction of Compound–Target–Pathway Network

According to the relationship between overlapping genes with ingredients and KEGG enrichment pathways, the compound–target–pathway network was constructed and visualized using Cytoscape 3.7.2.

### 4.9. RNA-Seq and Data Analysis

K562 cells were treated with GD-NAIs (200 μg/mL) for 48 h. The cells were collected, and total RNA was extracted using TRIzol reagent TRIzol^®^ Reagent (Invitrogen, USA) according to the instruction manuals. RNA quality and quantity were determined by 2100 Bioanalyzer (Agilent Technologies, CA, USA) and NanoDrop 2000 (Thermo Fisher Scientific, Wilmington, DE, USA), respectively. One microgram of total RNA was used to construct an RNA-seq transcriptome library using a TruSeqTM RNA Sample Preparation Kit from Illumina (San Diego, CA) based on the product instructions. The RNA-seq sequencing library was sequenced using the Illumina HiSeq xten/NovaSeq 6000 platform (2 × 150 bp read length). Library preparation and sequencing were performed by Shanghai Majorbio Biopharm Biotechnology Co., Ltd. (Shanghai, China). The raw paired-end reads were trimmed and quality controlled by SeqPrep (https://github.com/jstjohn/SeqPrep (accessed on 7 June 2022)) and Sickle (https://github.com/najoshi/sickle (accessed on 7 June 2022)) with default parameters. The clean reads were aligned to the genome using HISAT2 (http://ccb.jhu.edu/software/hisat2/index.shtml (accessed on 7 June 2022)) software [62]. The mapped reads were assembled using StringTie (https://ccb.jhu.edu/software/stringtie/index.shtml?t=example (accessed on 7 June 2022)) [63].

### 4.10. Differential Expression Analysis and Functional Enrichment

The expression of each transcript was normalized by transcripts per million (TPM). Gene abundance was quantified using RSEM (http://deweylab.biostat.wisc.edu/rsem/ (accessed on 15 June 2022)) [64]. Differentially expressed genes (DEGs) between the control and GD-NAIs-treated groups were screened by the following parameters: *p*-value < 0.05 and log2FC > 1.5. Gene Ontology (GO) enrichment analysis, KEGG pathway analysis and Reactome pathway enrichment analysis were carried out using Goatools (https://github.com/tanghaibao/Goatools (accessed on 29 June 2022)), KOBAS (http://kobas.cbi.pku.edu.cn/home.do (accessed on 29 June 2022)) and the Reactome Database (http://reactome.org/ (accessed on 30 June 2022)), respectively. Disease Ontology (DO) enrichment analysis was performed using the Disease Ontology database (http://disease-ontology.org (accessed on 30 June 2022)) [65].

### 4.11. Statistical Analysis

Data are presented as the mean ± standard deviation (SD). Differences between groups were analyzed by one-way univariate analysis of variance (ANOVA). A *p*-value < 0.05 was considered to be statistically significant (marked as *). Higher significance levels were established at *p*-value < 0.01 (marked as **) and *p*-value < 0.001 (marked as ***).

## 5. Conclusions

In conclusion, we revealed the chemical components of GD-NAIs, confirmed the anti-CML activity, and elucidated the associated underlying mechanism of GD-NAIs against CML by combining UHPLC-HRMS-MN, network pharmacology and RNA-seq. Seventy-five sesquiterpenoids were tentatively characterized in GD-NAIs, in which four sesquiterpenoids would be the main active ingredients against CML, regulating PD-L1 expression and the PD-1 checkpoint pathway in cancer, PI3K/AKT, JAK/STAT, TGF-β, estrogen, Notch and Wnt signaling pathways. Our study is meaningful for understanding the pharmacological activity of GD-NAIs and provides an alternative agent against CML.

## Figures and Tables

**Table 1:** Detailed information on tentatively characterized sesquiterpenoids within *Gynura divaricata* (L.) DC.

Compound	Molecular Formula	Molecular Ion	Retention Time (min)	Error (ppm)	Fragment Ions (*m*/*z*)	References
**1**	C_15_H_20_O_3_	249.1484	10.262	−0.5	231.1436, 203.1423, 175.1118, 161.0947, 157.1016, 133.1014, 119.0861, 105.0693, 91.0544, 81.0697, 79.0539	[27]
2 *	C_15_H_22_O_3_	251.1631	10.264	−4.3	233.1541, 205.1591, 177.1266, 131.0851, 105.0696	/
3 *	C_15_H_24_O_3_	253.1788	8.585	−4.0	217.1578, 177.1263, 133.1007, 119.0850, 105.0694	/
4 *	C_12_H_16_O_2_	193.1219	9.648	−2.1	147.1170, 133.1015, 119.0840, 105.0690	/
5 *	C_15_H_24_O	221.1898	12.214	−0.9	203.1736, 161.1327, 147.1157, 119.0846, 105.0694	/
6 *	C_12_H_18_O_3_	211.1325	9.607	−1.2	175.1098, 147.1166, 119.0850, 105.0688	/
7 *	C_14_H_18_O_2_	219.1373	10.458	−3.0	145.1006, 131.0843, 119.0852, 105.0691, 91.0539, 77.0391	/
8 *	C_18_H_26_O_3_	291.1955	11.031	0.1	245.1901,147.0808, 133.1008, 119.0847, 105.0698, 69.0692	/
**9**	C_15_H_22_O	219.1739	11.779	−3.4	161.0961, 105.0695	[28]
10 *	C_13_H_20_O_2_	209.1531	10.107	−2.4	191.1505, 147.1169, 133.1007, 105.0695	/
11 *	C_18_H_26_O	259.2059	14.156	1.0	199.1510, 173.1326, 131.0853, 105.0695, 91.0539	/
12 *	C_14_H_22_O	207.1737	10.085	−3.1	189.1274, 147.0812, 105.0697, 91.0542	/
13 *	C_13_H_18_O_2_	207.1378	9.958	−0.8	189.1270, 105.0697, 91.0540	/
14 *	C_13_H_20_O_3_	225.1487	8.473	0.8	165.1263, 147.1164, 133.1000, 105.0692, 91.0539	/
15 *	C_14_H_20_O_2_	221.1532	12.627	−1.8	161.1313. 147.1179, 133.1023, 119.0851, 105.0690	/
16 *	C_14_H_22_O_2_	223.1693	10.367	0.2	205.1580, 190.1361, 161.0962, 119.0846, 105.0706, 91.0535, 79.0557, 67.0544	/
17 *	C_18_H_26_O_2_	275.1996	12.681	−3.5	173.1320, 159.1161, 131.0852, 105.0693, 91.0539	/
**18**	C_15_H_22_	203.1791	9.823	−1.6	161.1317, 145.0994, 133.1016, 119.0845, 105.0695	/
19 *	C_19_H_30_O	275.2363	13.171	−0.5	199.1488, 173.1340, 159.1173, 133.1012, 119.0854, 105.0693	/
**20**	C_16_H_20_O_3_	261.1482	11.287	−1.2	229.1236, 201.1268, 187.1121, 147.0811, 105.0698, 91.0541	[29]
21 *	C_15_H_24_O_4_	269.1742	9.915	−2.0	233.1571, 215.1345, 119.0853, 105.0699	/
22 *	C_20_H_30_	271.2412	11.057	−3.1	215.1827, 159.1165, 145.1002, 133.1016, 119.0852, 105.0690	/
23 *	C_14_H_20_O	205.1582	15.026	−2.4	190.1352, 175.1107, 105.0695, 91.0535	/
24 *	C_13_H_14_O_3_	219.1007	9.609	−4.0	177.0539, 149.0589, 141.0700, 115.0532, 105.0699	/
25 *	C_16_H_28_O_2_	253.2153	9.538	−3.6	189.1270, 177.1274, 159.1167, 145.1010, 133.1005, 105.0696	/
26 *	C_14_H_20_O_4_	253.1428	10.433	−2.5	189.1267, 133.1010, 119.0848, 105.0696	/
**27**	C_15_H_22_O_2_	235.1688	7.907	−1.9	189.1260, 177.1272, 159.1157, 142.0774, 133.1009, 105.0695	/
**28**	C_15_H_20_O	217.1584	11.009	−1.3	159.0803, 145.0647, 141.0701, 119.0853, 105.0699	[30]
29 *	C_14_H_20_	189.1643	11.308	2.8	145.1002, 129.0695, 119.0850, 105.0687	/
30 *	C_12_H_16_O_3_	209.1168	9.778	−2.0	151.1111, 105.0687	/
31 *	C_13_H_14_O_2_	203.1063	13.237	−1.8	161.0964, 147.0798, 105.0699	/
32 *	C_13_H_16_O_3_	221.1162	9.961	−4.6	206.0918, 161.0944, 128.0612, 105.0691	/
**33**	C_15_H_22_O_4_	267.1592	7.603	0.8	249.1479, 189.1270, 161.0957, 105.0689	[31]
**34**	C_15_H_22_O_5_	283.1534	5.029	−2.1	265.1420, 247.1323, 235.1325, 189.1267, 105.0693	[32]
35 *	C_20_H_30_O_2_	303.231	13.414	−2.8	285.2205, 267.2112, 173.1315, 105.0696	/
36 *	C_15_H_18_O	215.1426	10.391	−2.1	185.0959, 167.0689, 145.0998, 129.0668, 115.0359, 105.0708	/
37 *	C_13_H_12_O_2_	201.0908	9.560	−1.0	129.0694, 115.0545, 105.0702, 91.0535	/
38 *	C_15_H_18_O	215.1427	11.434	−1.6	199.1099, 185.0961, 115.0540, 105.0698	/
39 *	C_15_H_18_O_2_	231.1377	11.625	−1.1	213.1222, 195.1155, 156.0938, 143.0857, 105.0698, 91.0540	/
40 *	C_14_H_18_O	203.1427	10.986	−1.7	174.0656, 157.1011, 141.0695, 128.0624, 115.0539, 105.0694	/
41 *	C_15_H_20_	201.1637	11.243	−0.4	173.1302, 145.1007, 105.0693	/
**42**	C_14_H_18_	187.1481	10.345	−0.1	157.1007, 145.1008, 91.0545	[33]
43 *	C_13_H_16_	173.1317	9.893	−0.1	157.0644, 143.0846, 115.0533, 105.0687	/
44 *	C_12_H_12_O	173.0959	10.455	−1.1	143.0856, 105.0697	/
45 *	C_10_H_16_O_3_	185.1174	10.304	1.0	91.0543, 79.0538	/
46 *	C_13_H_16_O	189.1271	7.903	−1.5	156.0941, 141.0696, 128.0615, 105.0713, 91.0543	/
47 *	C_15_H_18_O_3_	247.1326	9.406	−1.1	187.1121, 145.1008, 128.0628, 105.0684	/
48 *	C_15_H_20_O_2_	233.1533	9.898	−1.3	173.1322, 145.1009, 105.0705	/
**49**	C_11_H_16_O_2_	181.1219	11.453	−2.2	163.1114, 135.1163, 105.0702, 91.0540	[34]
50 *	C_20_H_34_O_3_	323.2573	13.420	−2.4	287.2362, 269.2266, 177.1264, 145.1004	/
51 *	C_18_H_24_O	257.1902	12.629	0.8	199.1520, 173.1322, 128.0623, 105.0702	/
52 *	C_19_H_30_O_3_	307.2268	10.024	0.1	179.0969, 165.1260, 119.0852, 105.0701	/
53 *	C_14_H_22_O_3_	239.1634	9.166	−3.2	193.1672, 161.0953, 145.1013, 105.0699, 91.0532	/
54 *	C_18_H_30_O_3_	295.2260	10.115	−2.6	259.2019, 179.1432, 151.1113, 105.0697	/
55 *	C_18_H_32_O_4_	313.2371	12.458	−0.8	233.1538, 201.1292, 133.1018, 105.0702	/
56 *	C_16_H_20_O_5_	293.1380	9.655	−1.2	257.1157, 173.1339, 149.0960, 145.0997, 105.0697	/
57 *	C1_8_H_28_O_3_	293.2103	14.021	−2.8	275.2009, 173.1320, 159.1171, 133.1008, 105.0699	/
58 *	C_19_H_32_O_3_	309.2414	10.632	−3.3	291.2270, 205.1568, 191.1436, 161.0951, 119.0848, 105.0702	/
59 *	C_18_H_30_O_4_	311.2212	9.305	−1.6	275.2006, 163.1111, 145.1008, 119.0858, 105.0695	/
60 *	C_10_H_16_O_2_	169.1223	8.886	−3.0	151.1131, 123.1164, 91.0538	/
**61**	C_12_H_18_O_2_	195.1375	13.301	−2.3	131.0857, 105.0697	[35]
62 *	C_10_H_16_O_3_	185.1174	10.304	1.0	97.0642, 91.0543, 79.0538, 69.0695, 55.0538	/
63 *	C_11_H_16_O_3_	197.1109	8.471	−1.6	161.0944, 133.1008, 105.0691, 91.0539	/
**64**	C_17_H_24_O_3_	277.1790	14.188	−3.0	203.1430, 189.1272, 175.1113, 135.1163, 105.0695	[36]
**65**	C_13_H_22_O_2_	211.1687	9.454	−2.6	133.1016, 105.0707	[37]
66 *	C_17_H_26_O_4_	295.1899	9.349	−1.6	247.1677, 119.0847	/
67 *	C_13_H_24_O_3_	229.1798	9.432	−0.1	149.1330, 131.1009, 121.1008, 105.0696, 91.0542	/
68 *	C_20_H_34_O_2_	307.2623	12.592	−2.8	289.2502, 187.1477, 161.1326, 147.1170	/
69 *	C_18_H_26_O_4_	307.1897	13.979	−2.2	207.1726, 161.1313, 149.1321, 105.0694	/
70 *	C_20_H_32_O	289.2522	9.255	−1.4	271.2408, 215.1786, 161.1322, 119.0848, 105.0692	/
71 *	C_20_H_32_O_3_	321.2420	11.191	−1.3	303.2310, 243.2109, 203.1786, 189.1640, 175.1477, 147.1167, 119.0852	/
**72**	C_21_H_36_O_6_	385.2579	15.588	−1.5	321.2033, 293.1721, 221.1901, 135.1154, 105.0695	[38]
73 *	C_22_H_40_O_5_	385.2937	16.090	−3.8	340.2603, 209.1631, 153.1260, 135.1168, 105.0702, 95.0858	/
74 *	C_20_H_32_O_2_	305.2469	9.411	−2.0	206.1653, 191.1429, 173.1327, 133.1009, 107.0855	/
75 *	C_20_H_30_O_3_	319.2258	12.104	−3.0	206.1662, 191.1428, 173.1327, 135.0799, 119.0850	/

* ingredients were identified for the first time.

## Data Availability

Data is contained within the article or supplementary material.

## Supplementary Materials

The following supporting information can be downloaded at: https://www.mdpi.com/article/10.3390/ph15111435/s1, Figure S1: Mass spectrum and proposed fragmentation pattern of compound **3**; Figure S2: Mass spectrum and proposed fragmentation pattern of compound **5**; Figure S3: Mass spectrum and proposed fragmentation pattern of compound **6**; Figure S4: Mass spectrum and proposed fragmentation pattern of compound **7**; Figure S5: Mass spectrum and proposed fragmentation pattern of compound **8**; Table S1: The absorption and drug-likeness parameters of 75 compounds in GD-NAIs; Table S2: 294 overlapping genes; Table S3: Detailed information of KEGG pathways which met the requirements of Gene count ≥10.

## Abbreviations

ABL1: Abelson murine leukemia; AEL: acute erythroid leukemia; BCR: breakpoint cluster region; CCK-8: Cell Counting Kit-8; CML: chronic myelogenous leukemia; DEGs: differential expression genes; DO: disease ontology; FPP: farnesyl pyrophosphate; FoxO: forkhead transcription factors of the class O; GO: gene ontology; GNPS: global natural products social; GD: *Gynura divaricata* (L.)) DC.; GD-E: the extracts of GD; GD-NAIs: the non-alkaline ingredients of GD; Hh: hedgehog; JAK/STAT: Janus kinase/signal transducer and activator of transcription; JNK: c-Jun NH(2)-terminal kinase; KEGG: Kyoto encyclopedia of genes and genomes; LSCs: leukemia stem cells; MAPK: mitogen-activated protein kinase; MN: molecular networking; MTT: thiazolyl blue tetrazolium bromide; PPAR: peroxisome proliferator-activated receptor; PBS: phosphate-buffer saline; PI3K/AKT: phosphoinositide 3-kinases/protein kinase B; PI: propidium iodide; PPI: protein–protein interaction; RNA-seq: RNA-sequencing; SPE: solid-phase extraction; TPM: transcripts per million; TGF-β: transforming growth factor-β; TK: tyrosine kinase; TKIs: tyrosine kinase inhibitors; UHPLC-HRMS: ultrahigh performance liquid chromatography–tandem high resolution mass spectrometry; VEGF: the vascular endothelial growth factor; Wnt: wingless-type MMTV integration site family.
