# Supplementary material for: Integrated Chemical Characterization, Network Pharmacology and Transcriptomics to Explore the Mechanism of Sesquiterpenoids Isolated from Gynura divaricata (L.) DC. against Chronic Myelogenous Leukemia
Source: Pharmaceuticals (Basel). 2022 Nov 19;15(11):1435. doi: 10.3390/ph15111435 (PMC9693606; doi:10.3390/ph15111435)
Supplement: Supplementary file 1 [file pharmaceuticals-15-01435-s001.zip › Supplementary Material-Figures.pdf]

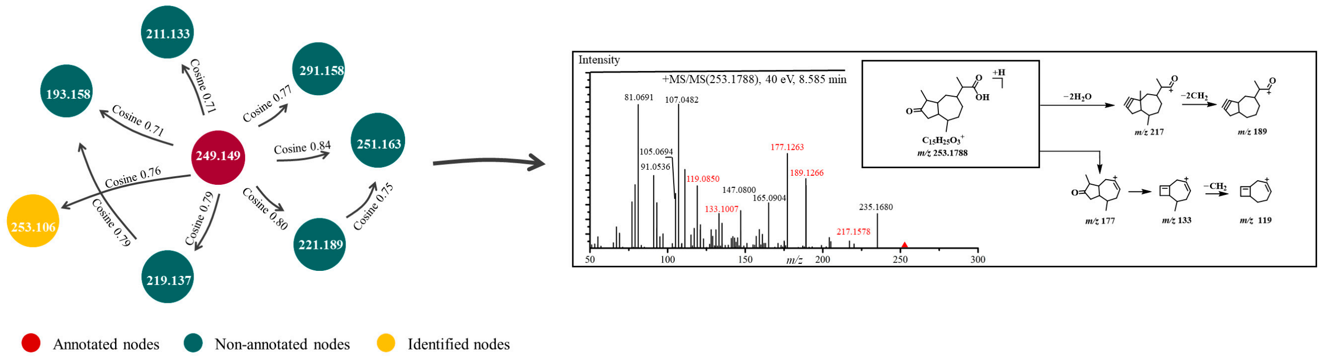

Figure S1. Mass spectrum and proposed fragmentation pattern of compound **3**.

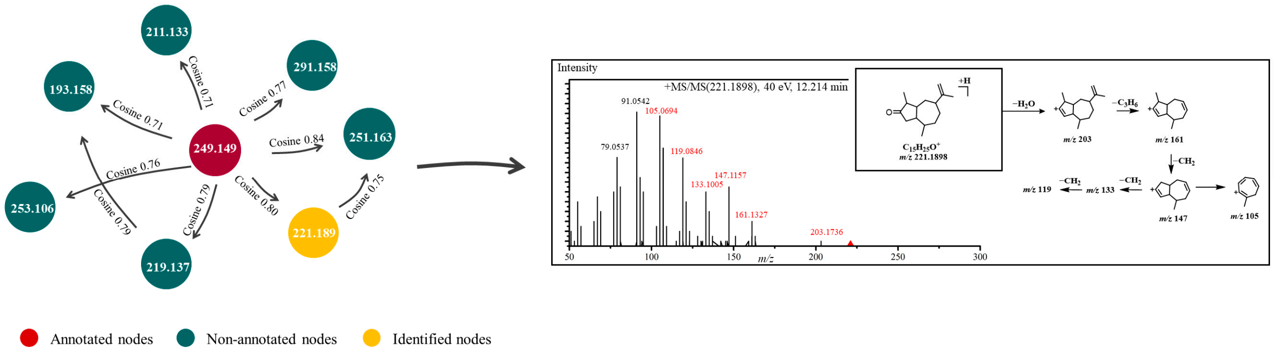

Figure S2. Mass spectrum and proposed fragmentation pattern of compound **5**.

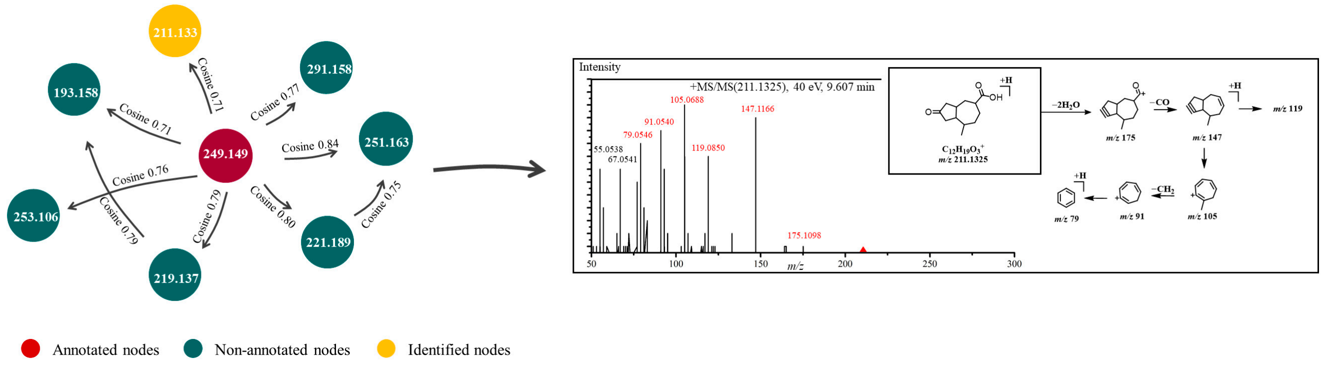

Figure S3. Mass spectrum and proposed fragmentation pattern of compound **6**.

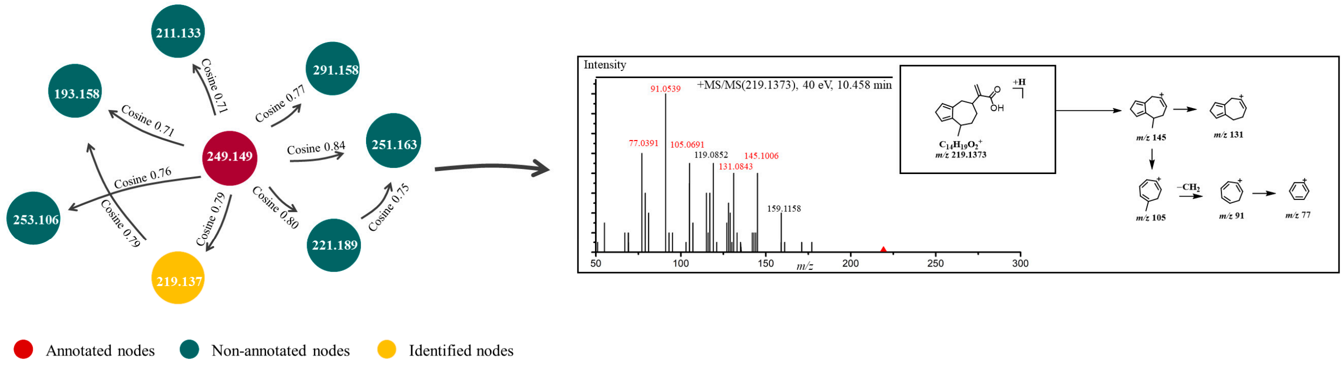

Figure S4. Mass spectrum and proposed fragmentation pattern of compound **7**.

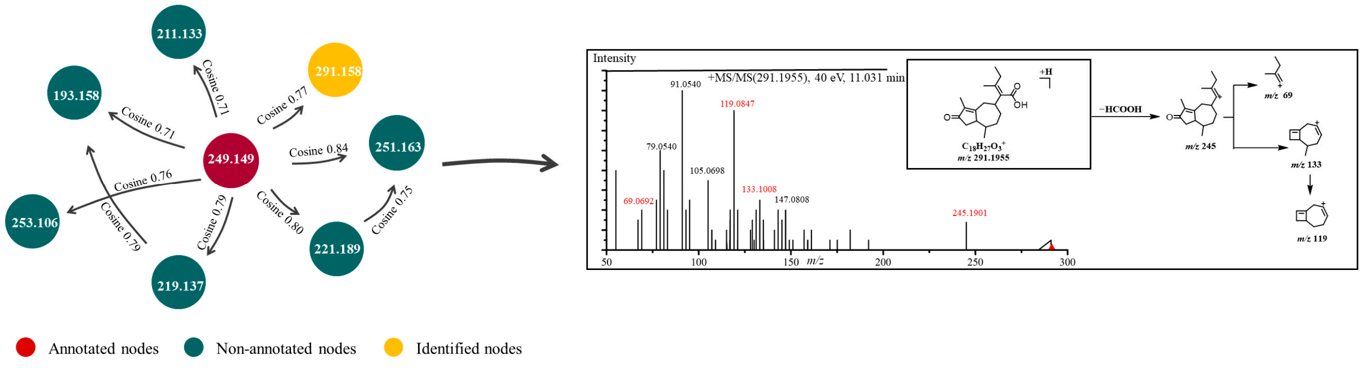

Figure S5. Mass spectrum and proposed fragmentation pattern of compound **8**.
